# miRNA Profiling Reveals Dysregulation of RET and RET-Regulating Pathways in Hirschsprung's Disease

**DOI:** 10.1371/journal.pone.0150222

**Published:** 2016-03-02

**Authors:** Shuangshuang Li, Shiqi Wang, Zhenhua Guo, Huan Wu, Xianqing Jin, Yi Wang, Xiaoqing Li, Shaoyan Liang

**Affiliations:** 1 Tumour laboratory of Children's Hospital of Chongqing Medical University, Ministry of Education Key Laboratory of Child Development and Disorders, Children's Hospital of Chongqing Medical University, Key Laboratory of Pediatrics in Chongqing, Children's Hospital of Chongqing Medical University, Chongqing International Science and Technology Cooperation Center for Child Development and Disorders, Chongqing 400014, PR China; 2 Department of Gastrointestinal Surgery and Neonatal Surgery, Children's Hospital of Chongqing Medical University, Chongqing 400014, PR China; University of Hong Kong, HONG KONG

## Abstract

Hirschsprung’s disease (HSCR), the most common congenital malformation of the gut, is regulated by multiple signal transduction pathways. Several components of these pathways are important targets for microRNAs (miRNAs). Multiple miRNAs have been associated with the pathophysiology of HSCR, and serum miRNAs profiles of HSCR patients have been reported, but miRNA expression in HSCR colon tissue is almost completely unexplored. Using microarray technology, we screened colon tissue to detect miRNAs whose expression profiles were altered in HSCR and identify targets of differentially expressed miRNAs. Following filtering of low-intensity signals, data normalization, and volcano plot filtering, we identified 168 differentially expressed miRNAs (104 up-regulated and 64 down-regulated). Fifty of these mRNAs represent major targets of dysegulated miRNAs and may thus important roles in the pathophysiology of HSCR. Pathway analysis revealed that 7 of the miRNA targets encode proteins involved in regulation of cell proliferation and migration via RET and related signaling pathways (MAPK and PI3K/AKT). Our results identify miRNAs that play key roles in the pathophysiology of the complex multi-factorial disease HSCR.

## Introduction

Hirschsprung's disease (HSCR) is a disorder of the abdomen that occurs when part or all of the large intestine or antecedent parts of the gastrointestinal tract have no ganglion cells and therefore cannot function. HSCR is a rare disease, occurring in approximately 1/5,000 live births [1]. Over the past decades, many studies have sought to elucidate the pathological network underlying this disease, but the detailed mechanism remains unknown. To date, more than 10 genes have been reported to play important roles in the development of HSCR, including *RET*, *GDNF*, *NRG1*, *EDNRB*, *SOX10*, *SIP1*, *PHOX2B and KIAA1279* [2–10]. Among these genes, receptor tyrosine kinase (RET) and glial cell line-derived neurotrophic factor (GDNF) the two major specific genes responsible for HSCR [11]. However, because of the complexity of the gnen regulatory involved, we are far from a full understanding of the pathology of HSCR. Discovery of miRNAs that target mRNAs encoding elements of the HSCR network would greatly expand our knowledge of the gene regulation throughout the development of this disease.

MicroRNAs (miRNAs), noncoding RNAs ~22 nucleotides in length, mediate silencing and post-transcriptional regulaton of gene expression [12, 13]. miRNAs play important roles in many vital processes, including cell differentiation, proliferation, migration and apoptosis [14]. miRNAs negatively regulate gene expression at the post-transcriptional level by interacting with the 3’ untranslated regions (3’-UTRs) of their target mRNAs [15]. Currently, more than 2500 human miRNAs are described in miRBase 21 [16–17], and more than 60% of protein-coding transcripts are predicted to be targets for regulation by miRNAs [18]. Some miRNAs can regulate large numbers of transcripts [19], and conversely, many mRNAs contain multiple miRNA binding sites [20]. Previous studies in human subjects demonstrated that miR-192/215, miR-206, miR-200a/141, miR-141 are down-regulated and miR218-1, miR-195 and miR-124 are markedly up-regulated, in stenotic colon segments relative to normal colon tissue [21–28]. These observation indicated that dysregulation of neuron cell migration and proliferative changes in stenotic colon segment of HSCR patients are linked to changes in miRNA expression levels. Microarray analysis of differentially expressed miRNAs in serum of HSCR patients identified several miRNAs as diagnositic markers of HSCR [29]. However, the involvement of miRNAs in HSCR patients is far from completely understood. Therefore, we performed a comprehensive microarray analysis of miRNA expression in colon tissue with the goal of identifying miRNAs that are differentially expressed in colon tissue during development of HSCR.

## Materials and Methods

### Sample gathering: patients and healthy controls

Colon tissue specimens were obtained from the Department of Pediatric Surgery, Chingqing Children's Hospital, with the approval of the Institutional Review Board of Children’s Hospital of Chongqing Medical University and with the written consent of all patients or legal gurdians. All experiments were in accordance with government policies and relevant guidelines. Samples from a total of 76 HSCR patients (39 males and 37 females) were collected at Chongqing Children’s Hospital from March 2013 to September 2013. HSCR patients were aged from 13 days to 4 years old and all were diagnosed by barium enema and anorectic manometer evaluation before surgical procedures and pathological analysis for definitive diagnosis. Three age-matched control colon tissues were collected from patients with colorectal trauma or undergoing anorectal colostomy at Chongqing Children’s Hospital. Full-thickness tissues were obtained and immediately stored in liquid nitrogen.

### miRNA microarrays

Microarray assays for miRNAs profiling were conducted by the Kangcheng Technology Co, Ltd (Shanghai, China). In total, 12 miRNA chips were prepared using miRNAs extracted from six stenotic colon segment samples, three control samples (anastomotic normal colon segments) from HSCR patients, and three normal colon tissues from subjects without HSCR. The microarray data is MIAME compliant (accession number: H1311006). To identify differentially expressed miRNAs with statistical significance, we performed filtering of low-intensity signals, normalization, quality assessment and volcano plot filtering on data from both groups (lesion and control). The criteria for up- or down-regulated miRNAs were as follows: |log2(fold change)| ≥ 1 and P-value≤0.05.

### miRNA target prediction

Focusing on the differentially expressed miRNAs, we predicted their putative mRNA targets considering only experimentally validated miRNA-mRNA interactions using the miRWalk software (http://www.umm.uni-heidelberg.de/apps/zmf/mirwalk/) [30]. Among the putative targets, only genes with previously reported functions in the pathology of Hirschsprung’s Disease were considered to represent true targets of miRNAs differentially expressed in HSCR. The GoGene (http://gopubmed.org/gogene) [31] and NCBI Pubmed databases (http://www.ncbi.nlm.nih.gov/pubmed/) were used for literature retrieval.

### Functional analyses

Functional analyses were performed using the DAVID software (https://david.ncifcrf.gov/home.jsp) [32, 33], which annotates the cellular, molecular, and biological interactions and functional properties of genes. DAVID functional analysis was carried out to identify biological processes significantly associated (P < 0.05, calculated using the right-tailed Fisher’s exact test) with miRNA-targeted mRNAs.

### Quantitative RT-PCR analysis of miRNAs targeting RET and related pathway

miRNA was extracted from HSCR stenotic colon segments and control colon tissue from 76 HSCR patients using SanPrep Column microRNA Mini-Preps Kit (Sangon Biotech, Shanghai, China). miRNAs were reverse transcribed into cDNA using the All-in-One™ miRNA First-Strand cDNA Synthesis Kit (GeneCopoeia Inc., Rockville, MD, USA). Realtime PCR was performed using the All-in-One™ miRNA qPCR Kit (GeneCopoeia). The primers were included in S1 Table and S2 Table.

## Results

### Identification of miRNAs differentially expressed in colonic lesions of HSCR patients

RNA was isolated from stenotic colon segments of HSCR patients (n = 6, three males and three female, labeled as 3, 4, 10, 13, 16, and 20), control colon segments from HSCR patients (n = 3, one female and two males, labeled as 3con, 4con, and 10con) and normal tissue from control subjects (n = 3, one males and two females, labeled as 2, 6, and 21).

**Fig 1:**
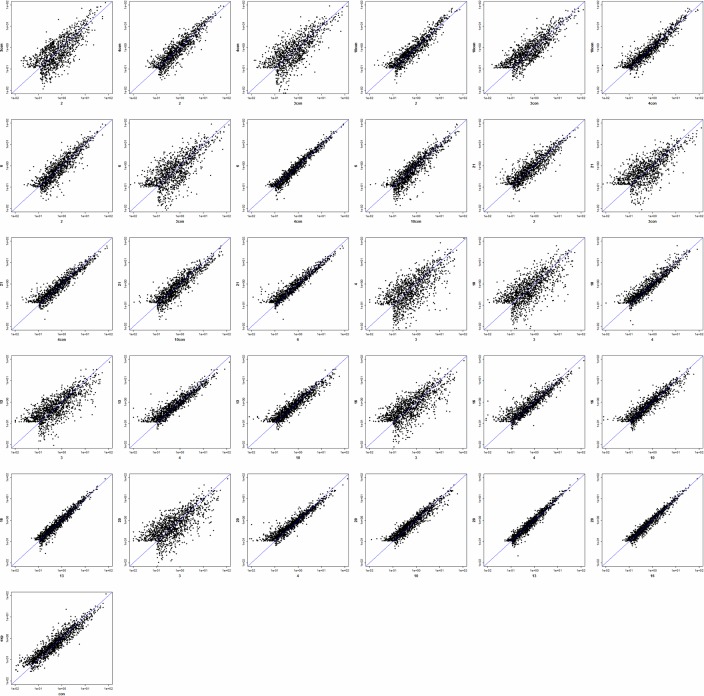
Scatter-plot to assess inter-chip variation. A scatter-plot was used to assess variation between chips. The axes of the scatter-plot show normalized signal values.

In totaly, 1,918 raw intensities were acquired. After filtering out the low-intensity signals, data normalization, and assessment of data quality after filtering, we obtained 290 miRNA expression data points. A correlation matrix (Table 1) and scatter-plot (Fig 1) were used to assess variation between samples; this assessment revealed a relatively good correlation between normal colon tissues and control colon tissues. Therefore, we pooled these two groups of tissues and labeled them as “normal”.

**Table 1:** Correlation coefficient matrix.

	2	3con	4con	10con	6	21
2	1	0.811644	0.933567	0.928441	0.926646	0.927493
3con	0.811644	1	0.802831	0.855139	0.790199	0.821824
4con	0.933567	0.802831	1	0.912029	0.980958	0.965628
10con	0.928441	0.855139	0.912029	1	0.899282	0.909014
6	0.926646	0.790199	0.980958	0.899282	1	0.971962
21	0.927493	0.821824	0.965628	0.909014	0.971962	1
	3	4	10	13	16	20
3	1	0.741872	0.735071	0.778827	0.776793	0.765929
4	0.741872	1	0.899341	0.944584	0.944766	0.965464
10	0.735071	0.899341	1	0.887571	0.901474	0.897829
13	0.778827	0.944584	0.887571	1	0.982051	0.983028
16	0.776793	0.944766	0.901474	0.982051	1	0.985639
20	0.765929	0.965464	0.897829	0.983028	0.985639	1
	con	exp				
con	1	0.896708				
exp	0.896708	1				

Table 1 shows the correlation matrix for the replicate samples used in this study.

**Fig 2:**
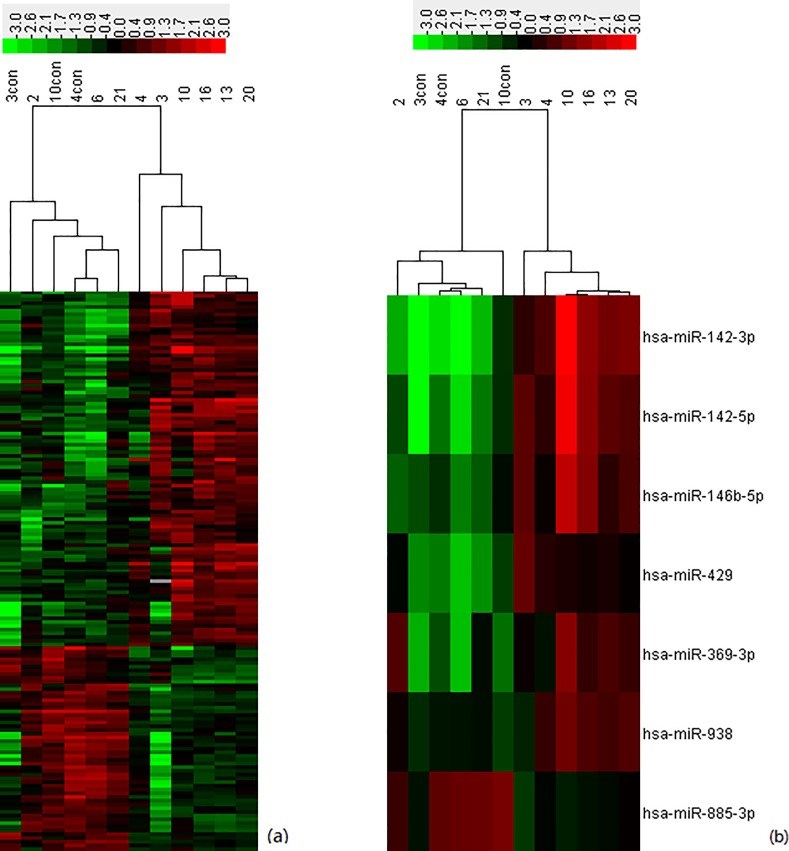
Heat map showing differentially expressed miRNAs. (a) Unsupervised hierarchic clustering of miRNAs differentially expressed between stenotic segments from HSCR patients and normal segments was performed using the Pearson correlation coefficient. Each row corresponds to one miRNA, and each column corresponds to one sample. Stenotic colon segments from HSCR patients are labeled as 3, 4, 10, 13, 16 and 20, control colon segments from HSCR patients are labeled as 3con, 4con, 10con and normal tissues from healthy subjects are labeled as 2, 6, and 21. (b) Unsupervised hierarchical clustering analysis of miRNAs targeting RET and RET-regulating pathways.

To identify miRNAs that were differentially expressed in a statistically significant manner, we performed volcano plot filtering between the two groups (HSCR and normals). The criteria for up- or down-regulated miRNAs were as follows: |log_2_(fold change)| ≥ 1 and P-value< = 0.05. In total, we identified 168 differentially expressed miRNAs (104 up-regulated and 64 down-regulated). Unsupervised hierarchic cluster analysis revealed that stenotic colon segment tissues could be distinguished from normal colon tissues based on their miRNA expression patterns (Fig 2A).

### Predicted targets of differentially expressed miRNAs affect cell proliferation through the RET pathway

Using the miRWalk software, we identified experimentally validated targets for differentially expressed miRNAs. After removing transcripts not associated with pathology of HSCR from the list of possible targets, we found that 13 of the 168 miRNAs targeted 50 potentially relevant mRNAs, whereas the other 155 miRNAs did not have any experimentally evaluated molecular targets associated with HSCR pathology (Tables 2 and 3).

**Table 2:** Summary of differentially expressed miRNAs.

	Normalized expression level			
miRNA ID	Mean of con group	Mean of exp group	-Fold change	P-value
**Up-regulated**				
hsa-miR-142-3p	1.552452	22.20496	14.30315	0.022786
hsa-miR-142-5p	0.434593	3.342842	7.691894	0.014472
hsa-miR-146b-5p	0.273015	1.11891	4.098344	0.008892
hsa-miR-338-3p	0.168553	0.592369	3.51444	1.45E-05
hsa-miR-369-3p	0.079203	0.184768	2.332848	0.040346
hsa-miR-429	0.212249	0.632613	2.980516	0.001719
hsa-miR-519b-3p	0.042262	0.096649	2.286893	0.002715
hsa-miR-614	0.086465	0.190521	2.203436	0.003912
hsa-miR-654-3p	0.061961	0.12517	2.020138	0.021811
hsa-miR-938	0.120295	0.245218	2.038465	0.004496
**Down-regulated**				
hsa-miR-107	7.237026	2.929471	0.404789	0.022436
hsa-miR-638	6.694976	3.012428	0.449953	0.002173
hsa-miR-885-3p	0.518701	0.226666	0.436987953	0.002046

miRNAs (differentially expressed [P ≤ 0.05, Student t test] in stenotic colon segment tissues versus normal colon tissues) that target experimentally validated mRNAs related to HSCR pathology.

**Table 3:** List of experimentally validated mRNA targets of differentially expressed miRNAs.

Gene symbol	Gene name	Targeting miRNA name
*ACE*	angiotensin I converting enzyme	hsa-mir-429
*ADAR*	adenosine deaminase, RNA-specific	hsa-mir-142-5p
*ADARB1*	adenosine deaminase, RNA-specific, B1	hsa-mir-142-5p
*AKT1*	v-akt murine thymoma viral oncogene homolog 1	hsa-mir-142-3p
*AMH*	anti-Mullerian hormone	hsa-mir-429
*APCS*	amyloid P component, serum	hsa-mir-142-5p
*BCL2*	B-cell CLL/lymphoma 2	hsa-mir-146b-5p
*BDNF*	brain-derived neurotrophic factor	hsa-mir-107
*BRCA1*	breast cancer 1, early onset	hsa-mir-369-3p,hsa-mir-146b-5p,hsa-mir-638
*CD4*	CD4 molecule	hsa-mir-614,hsa-mir-146b-5p,hsa-mir-142-3p, hsa-mir-142-5p
*CD68*	CD68 molecule	hsa-mir-146b-5p
*CD79A*	CD79a molecule, immunoglobulin-associated alpha	hsa-mir-429,hsa-mir-146b-5p
*CD8A*	CD8a molecule	hsa-mir-614,hsa-mir-146b-5p,hsa-mir-142-3p, hsa-mir-142-5p
*CDKN1A*	cyclin-dependent kinase inhibitor 1A (p21, Cip1)	hsa-mir-654-3p,hsa-mir-519b-3p,hsa-mir-338-3p,hsa-mir-146b-5p,hsa-mir-142-3p
*CDKN2A*	cyclin-dependent kinase inhibitor 2A	hsa-mir-885-3p
*CREB1*	cAMP responsive element binding protein 1	hsa-mir-142-3p
*CXCR4*	cell surface receptor for the CXC chemokine PBSF/SDF-1	has-mir-142-3p, has-146b-5p
*DMD*	Dystrophin	hsa-mir-146b-5p
*E2F1*	E2F transcription factor 1	hsa-mir-107
*EIF2C2*	argonaute RISC catalytic component 2	hsa-mir-107
*FGF2*	fibroblast growth factor 2	hsa-mir-146b-5p
*FOS*	FBJ murine osteosarcoma viral oncogene homolog	hsa-mir-146b-5p
*GLI1*	GLI family zinc finger 1	hsa-mir-429
*GLI3*	GLI family zinc finger 3	hsa-mir-429
*IHH*	indian hedgehog	hsa-mir-429
*IL1B*	interleukin 1, beta	hsa-mir-146b-5p, hsa-mir-142-5p
*IL6*	interleukin 6	hsa-mir-146b-5p
*ITGA3*	integrin, alpha 3	hsa-mir-142-3p
*JUN*	jun proto-oncogene	hsa-mir-146b-5p, hsa-mir-142-3p
*KIT*	v-kit Hardy-Zuckerman 4 feline sarcoma viral oncogene homolog	hsa-mir-107
*MAP2K1*	mitogen-activated protein kinase kinase 1	hsa-mir-146b-5p
*MAPK1*	mitogen-activated protein kinase 1	hsa-mir-146b-5p
*MAPK3*	mitogen-activated protein kinase 3	hsa-mir-146b-5p
*MECP2*	methyl CpG binding protein 2	hsa-mir-146b-5p
*MITF*	microphthalmia-associated transcription factor	hsa-mir-429
*MYCN*	v-myc avian myelocytomatosis viral oncogene neuroblastoma derived homolog	hsa-mir-429
*NF2 *	neurofibromin 2 (merlin)	hsa-mir-885-3p,
*PLCG1*	phospholipase C, gamma 1	hsa-mir-429
*POMC*	Proopiomelanocortin	hsa-mir-142-3p
*PTCH1*	patched 1	hsa-mir-146b-5p
*PTEN*	phosphatase and tensin homolog	hsa-mir-429
*RET*	receptor tyrosine kinase	hsa-mir-146b-5p
*SHH*	sonic hedgehog	hsa-mir-429
*SNAI2*	snail family zinc finger 2	hsa-mir-429
*SOX10*	SRY (sex determining region Y)-box 10	hsa-mir-338-3p
*TGFB1*	transforming growth factor, beta 1	hsa-mir-938,hsa-mir-429
*TNF*	tumor necrosis factor	hsa-mir-369-3p,hsa-mir-146b-5p,hsa-mir-142-5p
*TP53*	tumor protein p53	hsa-mir-429,hsa-mir-146b-5p,hsa-mir-142-3p
*ZEB1*	zinc finger E-box binding homeobox 1	hsa-mir-429
*ZEB2*	zinc finger E-box binding homeobox 3	hsa-mir-429

Fifty experimental valided miRNA targets associated with HSCR were identified using miRWalk software and GoGene and NCBI PubMed databases.

**Fig 3:**
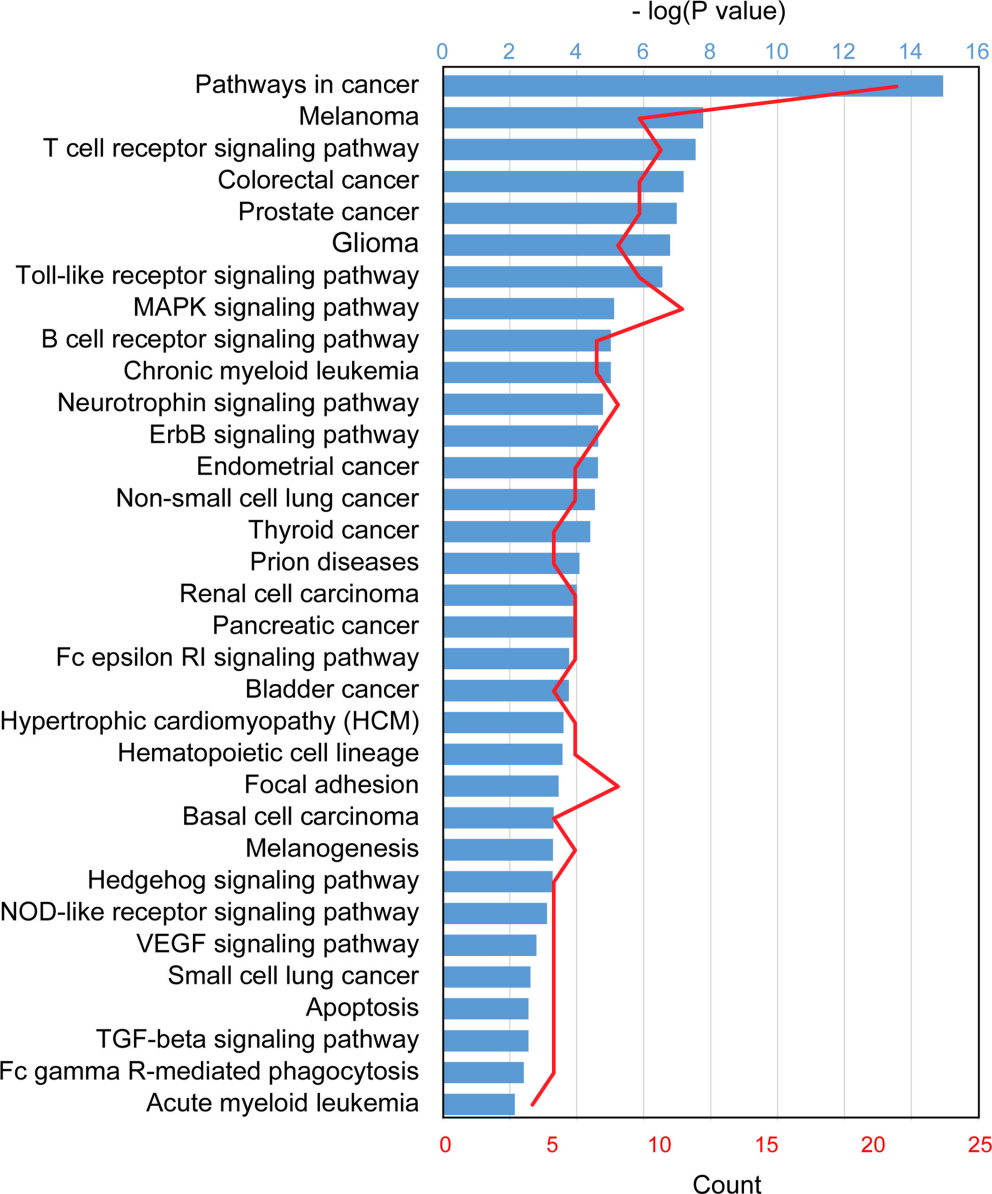
DAVID KEGG pathway analysis. KEGG pathway analysis of the list of 50 miRNA targets. The vertical axis provides the names of the most significantly overrepresented pathways (P < 0.01), whereas the horizontal axis shows the -2log_10_(P), where P was calculated based on Fisher’s exact test. The ratio (red) represents the numbers of genes in a given pathway that meet the cutoff criteria, divided by the total number of genes in that pathway.

To determine the gene regulatory pathways that could be affected by the differentially expressed miRNAs identified in this study, we performed DAVID functional analysis on a set of 50 mRNAs, 43 targeted by up-regulated miRNAs and seven targeted by down-regulated miRNAs, known or reasonably predicted to participate in development of HSCR. Most pathways identified by DAVID in this cluster of genes (Fig 3) are involved in processes related to cancer, including cell proliferation and migration (RET, the mitogen-activated protein kinase [MAPK] pathway, phosphatase and tensin homolog [PTEN], phosphoinositide 3-kinase [PI3K]/AKT, the hedgehog signaling pathway, p53, p21, and ZEB2), apoptosis (BCL-2, PTEN, PI3K/AKT, p53, p21, and c-Jun), and inflammation (TNFα, IL1β, IL-6, the MAPK pathway, and PI3K/AKT). RET and related signaling pathways (MAPK and PI3K/AKT) caught our attention because RET makes a major contribution to the pathology of HSCR, and silencing of RET leads to repression of cell proliferation and migration. Furthermore, transcripts of 14 genes involved in these pathways (RET, FGF, MAPK3, IL1B, JUN, MAPK1, MAP2K1, TGFB, TNF,TP53, AKT, FOS, p21, and PTEN) are among the 50 major targets of the 7 of the 13 differentially expressed miRNAs described above (hsa-miR-142-3p, hsa-miR-142-5p, hsa-miR-146b-5p, hsa-miR-369-3p, hsa-miR-429, hsa-miR-938, and hsa-miR-885-3p).

### Six of seven miRNAs targeting RET and its related signaling pathways are differentially expressed in stenotic segments versus control segments from HSCR pations

**Fig 4:**
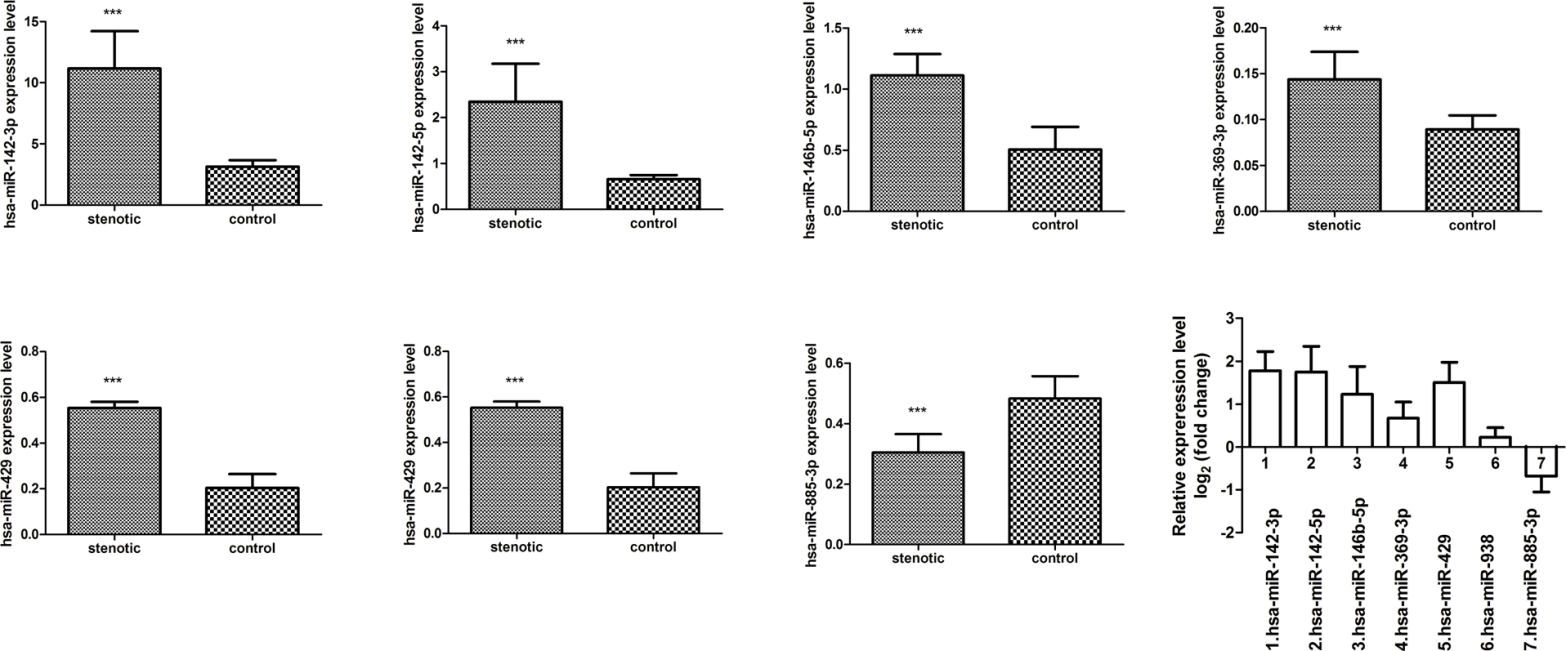
Quantitative RT-PCR analysis. Quantitative RT-PCR analysis of miRNAs targeting RET and its related pathways in76 stenotic segments and matched control tissue from HSCR patients, *, P < 0.05 versus control group, **, P < 0.01 versus control group.

To examine the validity of the seven potential miRNAs targeting RET and its relevant signaling pathways, we examined their expression levels in 76 stenotic segment tissues and matched control tissues from HSCR patients. Real-time PCR revealed that six of these miRNAs (hsa-miR-142-3p, hsa-miR-142-5p, hsa-miR-146b-5p, hsa-miR-369-3p, and hsa-miR-429) were significantly up-regulated in stenotic segments (P<0.05), whereas hsa-miR-885-3p was significantly down-regulated (P<0.05) (Fig 4). This observation suggests that these six miRNAs might be involved in the pathological development of HSCR.

### Decreased expression of RET members and molecules involved in related signaling pathways

**Fig 5:**
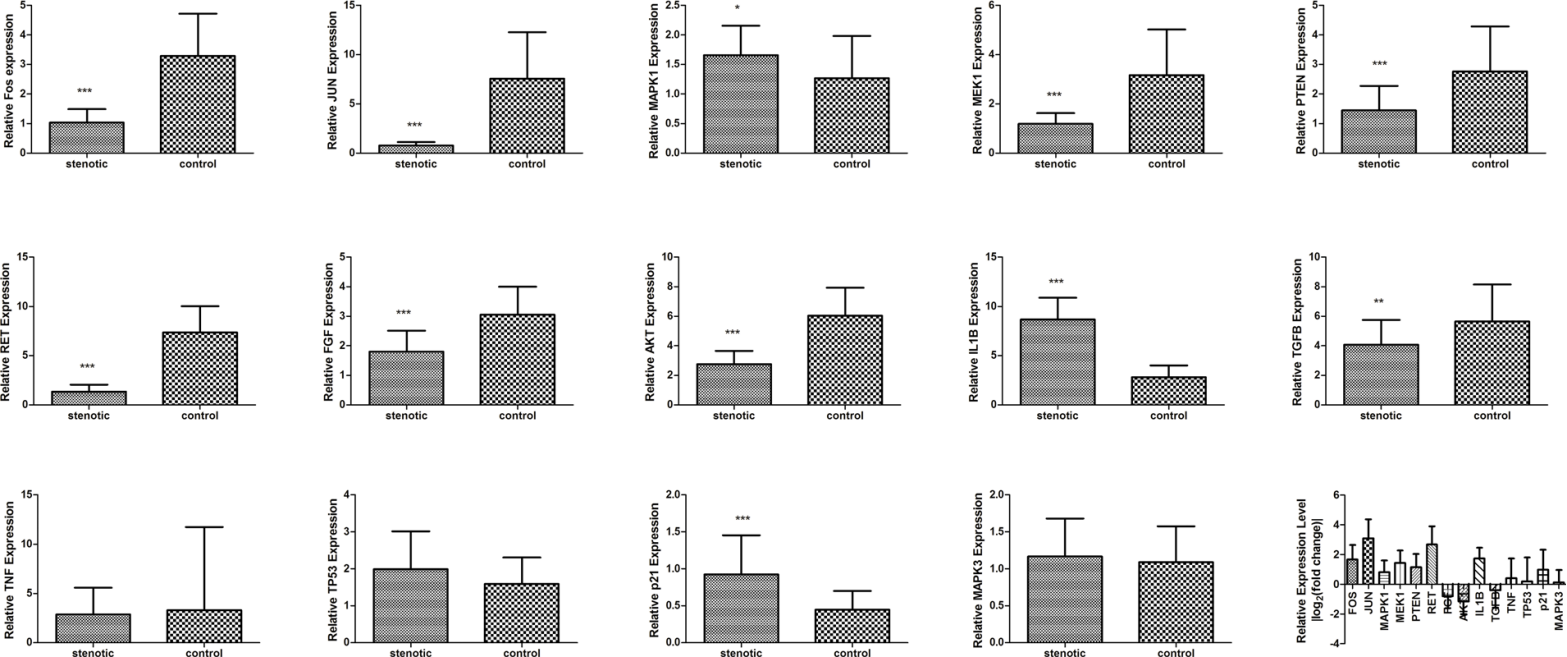
Changes in the expression of RET members and molecules associated with RET-related signaling pathways in stenotic and control colon tissues. Compared with control colon tissues, stenotic colon tissues showed significantly down-regulated expression of RET and molecules involved in RET-associated signaling pathways (*p< 0.05). Thirty-two stenotic and control colon tissues from patients with HSCR (aged 3 months to 4 years) were used for RNA extraction and qRT-PCR analysis.

To validate the accuracy of the miRNA analysis and their association with RET members and other molecules involved in related signaling pathways commonly found in stenotic and control tissues, we examined changes in the expression of such molecules in stenotic colon tissues. As shown in Fig 5, compared with control colon tissues, stenotic colon tissues showed significant downregulation of most members of RET and other molecules involved in RET-associated signaling pathways (p< 0.01) except TNF, TP53 and MAPK3.

## Discussion

miRNA expression levels change in many diseases, including HSCR, and play important roles in the pathogenesis. Discovery of miRNA expression patterns in HSCR might help to identify the complex regulatory network associated with this congenital disease, whose underlying pathology is still not completely understood. Previous work identified several miRNAs differentially regulated in the senotic tissue of HSCR patients [21–28]. Other studies sought to identify diagnostic bio markers for HSCR by analyzing miRNAs differentially expressed in the serum of the HSCR patients and control subjects [29].

However, the role of miRNAs in HSCR patients is far from fully elucidated. Therefore we performed a comprehensive microarray analysis of colon tissue with the goal of identifying miRNAs differentially expressed in colon tissue during HSCR development. We obtained normal colon tissues from otherwise healthy subjects who underwent anorectal colostomy or surgery for colorectal trauma whereas stenotic segments and control tissue samples were obtained from HSCR patients. We then evaluated the complete miRNA profiles of these tissues, providing the first comprehensive picture of miRNA dysregulation in HSCR colon tissue.

Microarray analysis revealed significant differences in miRNA expression patterns between HSCR stenotic colon segments and tissues from control tissue. Microarrays are sensitive and allow the detection of subtle changes in expression. Therefore, 15.1% of the expressed miRNAs identified herein exhibited a |log_2_(fold change)| ≥ 1 and a P-value <0.05. The maximum change in expression (14.3-fold) was observed for hsa-miR-142-3p.In total, we identified 168 miRNAs (104 up-regulated and 64 down-regulated) that were differentially expressed in a statistically significant manner (|log_2_(fold change)| ≥ 1, P ≤ 0.05) between these two types of sample.

**Fig 6:**
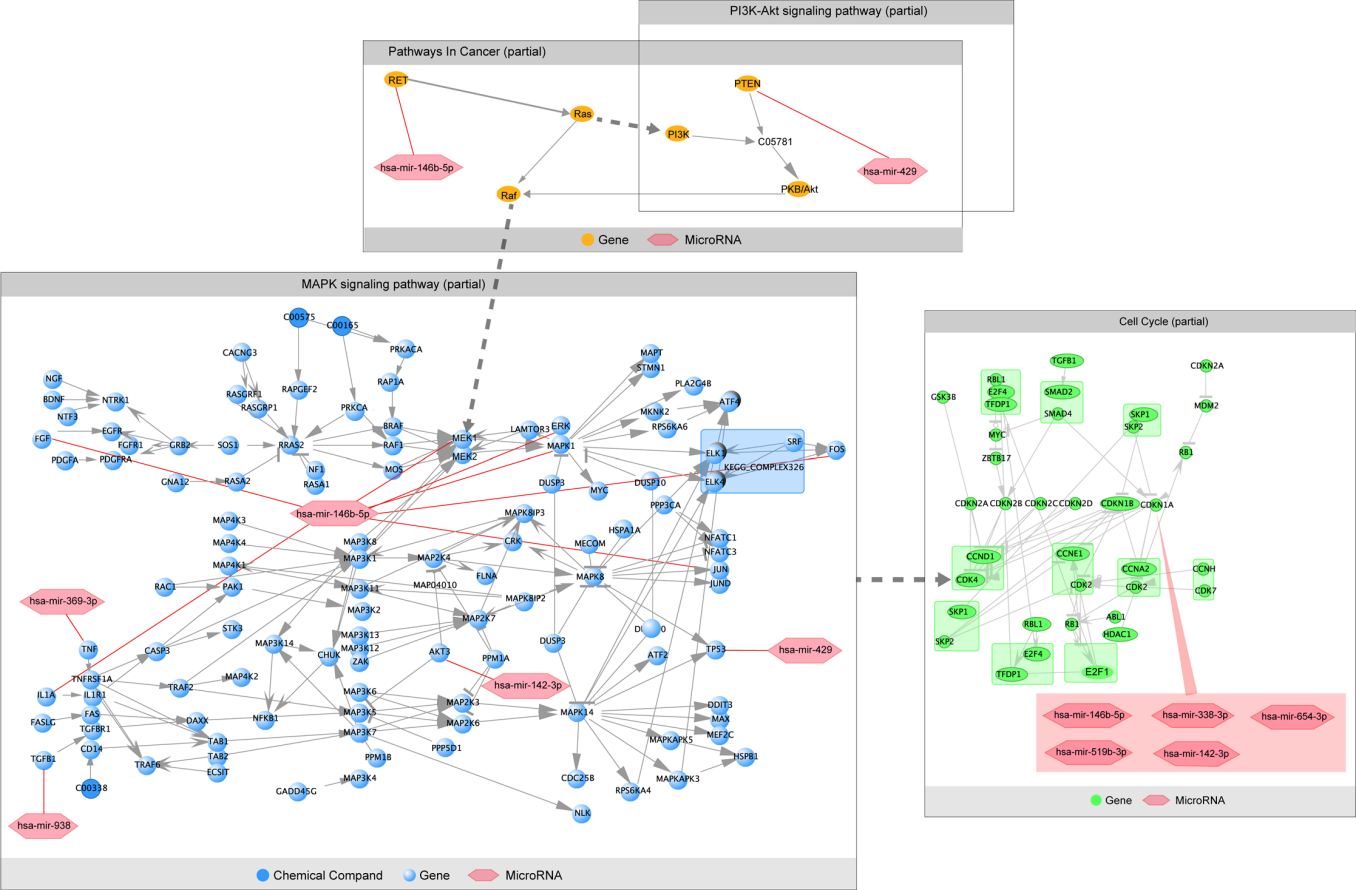
Deregulation of RET and its regulating signaling pathways in HSCR patients. Genes are shown in the chart in round shape. miRNAs are shown in the chart in hexagon. Continuous or dashed lines stand for direct or indirect relationship.

miRNAs regulate gene expression by base paring partially complementary binding sites in the 3’-UTRs of their mRNA targets, lresulting in translational silencing or mRNA degradation [34]. Analysis of experimentally validated mRNA targets of the miRNAs differentially expressed in stenotic colon segments of HSCR patients allowed us to identify a set of 50 mRNAs representing the major targets of these miRNAs (Table 3). To further explore the biological and functional roles of the differentially expressed miRNAs, we identified the most relevant regulatory pathways associated with their target genes. This analysis revealed that many key cellular signaling pathways could be perturbed by changes in miRNA expression (Fig 4). Among them, RET attracted our attention because it encodes proteins that help neural crest cells to move through the digestive tract during the development of the embryo, and is a major contributor to the pathology of HSCR [35]. Furthermore, RET can autophosphorylate and activate downstream signaling programs, such as the MAPK and PI3K/ATK pathways [36], which influence enteric neural crest stem cells (ENCC) proliferation and survival, apoptosis, migration, and differentiation; these pathways are most likely to be affected by HSCR-related changes in miRNA expression [37]. Additionally, six of the markedly changed miRNAs (validated in stenotic segments and control tissue samples from HSCR patients) targeted RET members and molecules associated with RET-related signaling pathways (i.e., MAPK and PI3K/AKT) (Fig 6). As many as 14 genes that participate in these pathways (*RET*, *FGF*, *MAPK3*, *IL1B*, *JUN*, *MAPK1*, *MAP2K1*, *TGFB*, *TNF*, *TP53*, *AKT*, *FOS*, *p21*, and *PTEN [38–52]*) belong to the list of 50 major experimentally validated targets for the differentially expressed miRNAs identified herein. Unsupervised hierarchical clustering analysis of these miRNAs revealed that the miRNA expression pattern could clearly distinguish stenotic colon segments obtained from HSCR patients from normal colon tissue obtained from healthy subjects (Fig 2B). We found a significant reduction in the expression of RET members and molecules involved in RET-related signaling pathways (Fig 5). We also found that hsa-miR-142-3p, hsa-miR-142-5p, hsa-miR-146b-5p, hsa-miR-338-3p, hsa-miR-369-3p, hsa-miR-429, and hsa-miR-519b-3p were significantly up-regulated in stenotic segments, whereas hsa-miR-107 and hsa-miR-638 were significantly down-regulated. This strongly supports our hypothesis that miRNA profiling can identify dysregulation of RET and RET-regulating pathways in Hirschsprung’s disease. Furthermore, preliminary data from our ongoing functional studies based on manipulated cell culture systems reveal that these miRNAs have the potential to act either individually or synergistically (Li and Wu, unpublished data), thereby confirming their role in HSCR. Taken together, these observations support the idea that the miRNA-regulated genes we identified play roles in HSCR pathophysiology.

## Conclusions

This study is the first comprehensive analysis of miRNA expression in colon tissue from HSCR patients, and the results revealed significant changes of several of these regulatory molecules in HSCR pathology. Based on these observations, we identified several mRNAs targeted by the differentially expressed miRNAs that belong to signaling pathways involved in HSCR pathology. We believe that the resultant lists of miRNAs and their targets provide new information that will be useful for understanding the molecular mechanisms underlying this complex multi-factorial disease and may also have important implications for prenatal diagnosis and therapeutics.

## Supporting Information

S1 Table Primer sequences for miRNA qRT-PCR. (DOC)

S2 Table Primers sequences for target gene qRT-PCR. (DOC)

## Abbreviations

HSCR: Hirschsprung’s disease
miRNAs: microRNAs
RET: Receptor tyrosine kinase
MAPK: Mitogen-activated protein kinase
PI3K: Phos-phatidylinositide-3kinase
Akt: v-akt murine thymoma viral oncogene
PCR: Polymerase Chain Reaction
ENCC: Enteric neural crest stem cells
