# Supplementary material for: miRNA Profiling Reveals Dysregulation of RET and RET-Regulating Pathways in Hirschsprung's Disease
Source: PLoS One. 2016 Mar 2;11(3):e0150222. doi: 10.1371/journal.pone.0150222 (PMC4774952; doi:10.1371/journal.pone.0150222)
Supplement: S1 Table — (DOC) [file pone.0150222.s001.doc]

Sense and anti-sense primers for miRNAs were as follows:

| **miRNA** | **Primer** | |
| --- | --- | --- |
| hsa-mir-142-3p | forward | 5’-ACACTCCAGCTGGGTGTAGTGTTTCCTACTT-3’ |
| hsa-mir-142-5p | forward | 5’-ACACTCCAGCTGGGCATAAAGTAGAAAG-3’ |
| hsa-mir-146-5p | forward | 5’-ACACTCCAGCTGGGTGAGAACTGAATTCCA -3’ |
| hsa-miR-338-3p | forward | 5’-ACACTCCAGCTGGGTCCAGCATCAGTGATT-3’ |
| hsa-miR-369-3p | forward | 5’ ACACTCCAGCTGGGAATAATACATGGTTG-3’ |
| hsa-mir-429 | forward | 5’-ACACTCCAGCTGGGTAATACTGTCTGGT-3’ |
| hsa-miR-519b-3p | forward | 5’- ACACTCCAGCTGGGAAAGTGCATCCTTTTA-3’ |
| hsa-miR-614 | forward | 5’- ACACTCCAGCTGGGGAACGCCTGTTCTTGCC-3’ |
| hsa-miR-654-3p | forward | 5’-ACACTCCAGCTGGGTATGTCTGCTGACCAT-3’ |
| hsa-miR-938 | forward | 5’- ACACTCCAGCTGGGTGCCCTTAAAGGTGAA-3’; |
| hsa-miR-107 | forward | 5’- ACACTCCAGCTGGGAGCAGCATTGTACAGGG-3’ |
| hsa-miR-638 | forward | 5’- ACACTCCAGCTGGGAGGGATCGCGGGCGGGTGG-3’ |
| hsa-miR-885-3p | forward | 5’-ACACTCCAGCTGGGTCCATTACACTACCCT-3’ |
|  | universal reverse | 5’-TGGTGTCGTGGAGTCG-3’ |
| U6 | forward | 5’-CTCGCTTCGGCAGCACA-3’ |
|  | reverse | 5’-AACGCTTCACGAATTTGCGT-3’ |
