# Supplementary material for: miRNA Profiling Reveals Dysregulation of RET and RET-Regulating Pathways in Hirschsprung's Disease
Source: PLoS One. 2016 Mar 2;11(3):e0150222. doi: 10.1371/journal.pone.0150222 (PMC4774952; doi:10.1371/journal.pone.0150222)
Supplement: S2 Table — (DOC) [file pone.0150222.s002.doc]

Sense and anti-sense primers for target genes are as follows:

| **Gene** | **Primer** | |
| --- | --- | --- |
| RET(human） | Forward | GTCCTCTTGCTCCACTTCAACG |
|  | Reverse | CCTGGCAGTTTTCCACACAGAC |
| PTEN（human) | Forward | TGAGTTCCCTCAGCCGTTACCT |
|  | Reverse | GAGGTTTCCTCTGGTCCTGGTA |
| MEK1（human） | Forward | GGTGTTCAAGGTCTCCCACAAG |
|  | Reverse | CCACGATGTACGGAGAGTTGCA |
| MAPK1(human) | Forward | ACACCAACCTCTCGTACATCGG |
|  | Reverse | TGGCAGTAGGTCTGGTGCTCAA |
| FOS(human) | Forward | GCCTCTCTTACTACCACTCACC |
|  | Reverse | AGATGGCAGTGACCGTGGGAAT |
| JUN(human) | Forward | CCTTGAAAGCTCAGAACTCGGAG |
|  | Reverse | TGCTGCGTTAGCATGAGTTGGC |
| p21(human) | Forward | AGGTGGACCTGGAGACTCTCAG |
|  | Reverse | TCCTCTTGGAGAAGATCAGCCG |
| TP53(human) | Forward | CCTCAGCATCTTATCCGAGTGG |
|  | Reverse | TGGATGGTGGTACAGTCAGAGC |
| TNF(human) | Forward | CTCTTCTGCCTGCTGCACTTTG |
|  | Reverse | ATGGGCTACAGGCTTGTCACTC |
| TGFB(human) | Forward | TACCTGAACCCGTGTTGCTCTC |
|  | Reverse | GTTGCTGAGGTATCGCCAGGAA |
| MAPK3(human) | Forward | TGGCAAGCACTACCTGGATCAG |
|  | Reverse | GCAGAGACTGTAGGTAGTTTCGG |
| IL1B(human) | Forward | CCACAGACCTTCCAGGAGAATG |
|  | Reverse | GTGCAGTTCAGTGATCGTACAGG |
| AKT1(human) | Forward | TGGACTACCTGCACTCGGAGAA |
|  | Reverse | GTGCCGCAAAAGGTCTTCATGG |
| FGF(human) | Forward | CATCAACGCCACCTACAAGGTG |
|  | Reverse | CTTGCACTGGAAGGACGTGGTC |
